# Recent Trends in SERS-Based Plasmonic Sensors for Disease Diagnostics, Biomolecules Detection, and Machine Learning Techniques

**DOI:** 10.3390/bios13030328

**Published:** 2023-02-27

**Authors:** Reshma Beeram, Kameswara Rao Vepa, Venugopal Rao Soma

**Affiliations:** Advanced Centre of Research in High Energy Materials (ACRHEM), DRDO Industry Academia—Centre of Excellence (DIA-COE), University of Hyderabad, Hyderabad 500046, Telangana, India

**Keywords:** biosensing, SERS, plasmonics, disease diagnosis, biomolecules, microorganisms, COVID-19, biohazardous molecules, cancer

## Abstract

Surface-enhanced Raman spectroscopy/scattering (SERS) has evolved into a popular tool for applications in biology and medicine owing to its ease-of-use, non-destructive, and label-free approach. Advances in plasmonics and instrumentation have enabled the realization of SERS’s full potential for the trace detection of biomolecules, disease diagnostics, and monitoring. We provide a brief review on the recent developments in the SERS technique for biosensing applications, with a particular focus on machine learning techniques used for the same. Initially, the article discusses the need for plasmonic sensors in biology and the advantage of SERS over existing techniques. In the later sections, the applications are organized as SERS-based biosensing for disease diagnosis focusing on cancer identification and respiratory diseases, including the recent SARS-CoV-2 detection. We then discuss progress in sensing microorganisms, such as bacteria, with a particular focus on plasmonic sensors for detecting biohazardous materials in view of homeland security. At the end of the article, we focus on machine learning techniques for the (a) identification, (b) classification, and (c) quantification in SERS for biology applications. The review covers the work from 2010 onwards, and the language is simplified to suit the needs of the interdisciplinary audience.

## 1. Introduction

Plasmonics is the study of electron oscillations in metal nanostructures and their interaction with electromagnetic radiation. Since its conception in the 1950s, researchers have been interested in studying the fundamentals of the effects of shape, surrounding medium, material, and their interaction with light of different wavelengths [1]. With this well-established knowledge, plasmonics is witnessing an enormous potential for applications in different fields, including forensics [2]; environmental safety [3]; biosensing [4,5,6,7,8,9,10,11], e.g., SARS-CoV-2 detection [12]; and homeland security [13]. The applications of plasmonics majorly rely on surface plasmon resonance (SPR) or localized surface plasmon resonance (LSPR) effects [14]. Some of the significant techniques that were developed using these include higher-order harmonic generation, microscopy, drug delivery, photovoltaics, surface-enhanced Raman spectroscopy (SERS) and fluorescence, and surface-enhanced infrared absorption spectroscopy (SEIAS) and waveguides. The use of plasmonics in these techniques has significantly improved their efficiency over existing conventional techniques, offering flexibility, signal enhancement, and ease of use [15]. Advents in plasmonics have led to the emergence of SERS with impressive signal enhancements over traditional Raman spectroscopy [16]. SERS-based sensing is being widely used for the trace detection of different molecules, such as explosives [17], pesticides [18,19], food adulterants [20,21], drugs [22], biomolecules [23,24,25,26,27], medicine [28,29,30], and microorganisms [31].

**Figure 1:**
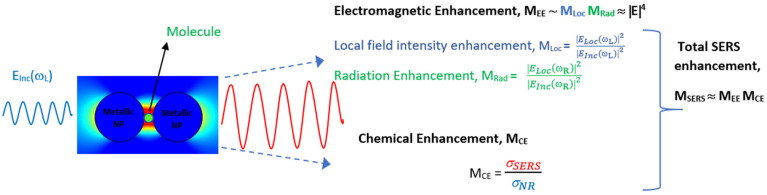
Schematic of total enhancement in SERS via electromagnetic and chemical enhancement mechanisms.

SERS typically utilizes localized surface plasmon resonances in metal nanostructures to enhance the weak Raman signal significantly. The phenomenon was first observed by Fleischmann in 1974 while studying pyridine adsorbed on a roughened silver electrode [32]. However, the enhancement was attributed to increased surface area for adsorption. It took further experiments in 1977 by two independent groups, Jeanmaire and van Duyne [33] and Albrecht and Creighton [34], to understand the origin of the enhancement. Now it is established that the enhancement predominantly comes from two mechanisms: electromagnetic enhancement (EE) and chemical enhancement (CE) [35]. The electromagnetic enhancement in SERS is a two-step process, and the total enhancement is multiplicative. When a molecule of interest is in the vicinity of a plasmonic nanostructure, it experiences an enhanced field called local field enhancement (LFE). The molecule then radiates with increased efficiency, referred to as radiation enhancement [36,37]. In addition, there is chemical enhancement which occurs because of charge-transfer mechanisms between nanoparticles and the analyte. Figure 1 summarizes the two enhancement mechanisms in SERS. The type of the plasmonic material, choice of wavelength, surface coverage of the molecules, and concentration of the analyte are the factors that influence SERS’s efficiency [38]. This technique is label-free, rapid, non-destructive, and water compatible and offers the fingerprint of the molecule, making it suitable for numerous applications. Nobel metals such as Au, Ag, and Cu and their alloys are the widely used materials for SERS for their tunability in the visible and IR region, inertness, sensitivity, and compatibility [39,40]. Despite the superior performance of Ag owing to its high-quality resonance in the visible region, Au is the preferred material, as it is known to be biocompatible and non-reactive in an oxygen atmosphere. The near-field enhancement in SERS is dependent on the shape and size of the nanostructures, in addition to the distance between the nanoparticles and distribution of probe molecules around the nanoparticles [41]. The different morphologies of nanoparticles, such as core–shell, rods, spherical, triangular, stars, and nanopyramids, are synthesized by widely reported chemical routes in bottom-up or top-down approaches [42]. Anisotropic nanostructures such as dendrites, rods, stars, and triangle are considered highly desirable for SERS since they enable lower detection limits owing to the lightening-rod effect [43,44]. The performance of SERS is also dependent on the choice of wavelength, and most biological tissues are transparent in the IR region, making it a preferred choice [45]. Recently, there is also growing interest in the UV and deep UV SERS for applications concerning biomolecules such as amino acids and DNA bases because they have electronic transitions in the UV region [39]. 

With a growing population and, consequently, the diseases worldwide, there is a need to develop point-of-care (POC) devices that are easy to use, reliable, rapid, and low cost. Over the years, SERS has been proven to possess all of these advantages, including trace detection with sub-picomolar sensitivity. Particularly, there are many reasons for the surge of using SERS for biosensing. Firstly, given the low scattering cross-section of water, SERS is extremely compatible with liquid samples, paving the way for use in biology applications, including liquid biopsy [46,47]. SERS has been widely used for disease diagnosis using urine, blood, serum, plasma, saliva, breath, and tear samples, establishing its compatibility. Measurements in SERS can be performed using liquids, gases, solids, and powders, unlike traditional tests. Secondly, SERS gives specific molecular information, which is often a vibrational fingerprint of the molecule or cell under study. Biomarkers that are Raman active are extensively used for the identification of different diseases, using SERS [48]. Frequently, when the variations are unrecognizable to the human eye, machine learning techniques are used to extract the patterns and discriminate the samples [49]. This was successfully used to classify normal and cancer cells [50], identify microorganism species [51], and monitor disease progression [52]. Thirdly, SERS is a rapid technique, can accomplish trace detection, and has a test time of three to five minutes [53]. Combined with recent developments in flexible SERS sensors, it also offers easy sample-collection methods, such as swabbing from an uneven surface [54]. Lastly, advances in portable instrumentation and low-cost lasers leveraged the usage of SERS for real-world applications [55]. The easy availability of IR lasers that have a low damage threshold with biology samples, as well as quench fluorescence, has favored the development of SERS for biosensing. All of these advantages have made SERS a popular choice for biosensing recently.

**Figure 2:**
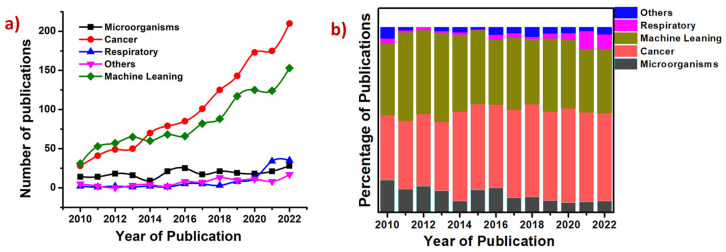
(**a**) Trends in research on SERS based plasmonic applications in the detection of microorganisms, cancers, respiratory diseases, other diseases such as heart ailments and diabetes and the use of different machine learning techniques for SERS based biosensing. (**b**) Bar chart with percentage contribution from each area shown on the label for the past 12 years. Source: Scopus search with the keywords mentioned in both the panels as on 5 January 2023.

There have been many review articles concerning the applications of plasmonics for biosensing and biosensors over the years. Salazar et al. and Han et al. reviewed different techniques, including LSPR, Chiral Plasmonic Biosensors, Magnetoplasmonic Biosensors, and Quantum Plasmonics Biosensors [56,57]. Anand et al. published a comprehensive review on plasmonic biosensors for the detection of viruses, with a special focus on COVID-19. They have focused on LSPR, SPR, SERS, SEF, and SEIAS techniques [58]. There are reviews and book chapters elaborating specifically on various SPR [59,60,61] and LSPR [62] techniques that are currently being used for biosensing. Similarly, Sarah et al. focused exclusively on LSPR techniques and associated challenges in the detection [63]. Alexandre reviewed the future of plasmonic biosensing with a goal of single-molecule and single-particle sensing [64]. Juanjuan et al. discussed on the challenges and future of using plasmonic materials for point-of-need applications [65]. However, although significant work has been performed using SERS for biology applications, no reviews of the literature can be found in this area. Here, we present a review of the work conducted in SERS for biosensing and the recent developments, with a special focus on machine learning techniques that are being used for the same. The article covers work from 2010 onwards and is organized into different sections, as shown in the index. Figure 2a,b illustrate the statistics of publications in different areas discussed in this review article. The data presented in the figure indicate that there is a growing interest in the usage of SERS for cancer-related applications and the usage of machine learning techniques for biosensing using SERS. There is also relative growth in using SERS for respiratory disease diagnosis recently owing to the COVID-19 situation.

## 2. SERS for Disease Diagnosis

With growing zoonotic diseases, cancers, diabetes, and other ailments, there is a pressing need to develop low-cost and POC identification techniques. Early and rapid diagnosis is the key to saving a life and prevent the rapid transmission of diseases. A trace detection technique such as SERS will aid in tracking the minute changes in cells or biomarkers, thus enabling early diagnosis. SERS is being extensively used for the same in both labeled and label-free approaches, often targeting specific biomarkers of the disease expression [30]. In the label-free approach, the sample is directly studied in contact with the plasmonic material, whereas in the labeled approach, a Raman reporter, such as fluorophores, antibodies, or ligands, is attached to the sample for detection and imaging [66,67]. Different biomarkers, such as proteins, antibodies, miRNAs, exosomes, and DNA, are used as indicators for the presence of the disease. In our observation, where full cells, tissues, or body fluids are studied, a machine learning algorithm is used hand in hand for accurate identification. SERS has been used for the detection of conditions such as Alzheimer’s [68,69,70,71], PCOS [72], diabetes [73,74], inflammation [74], Crohn’s disease [75], and single Hb molecule [76], to name a few. Here we review the progress on SERS for the diagnosis of (a) cancer, paying special attention to lung and breast cancer, as they are the leading causes of deaths due to cancer; and (b) respiratory viruses, including COVID-19.

### 2.1. Cancer Diagnosis and Theranostics

Cancer is the new pandemic and a leading cause of deaths in the modern world [77]. There is an increase in the incidence of various types of cancers, including mouth, gastric, lungs, ovaries, skin, and blood cancer. Numerous factors, such as environment, diet, lifestyle, and smoking, can trigger cancer. The early diagnosis of cancer is extremely important, as it is lifesaving with existing treatment protocols. Conventional cancer diagnosis is often performed using imaging techniques such as X-ray, computerized tomography scan (CT), positron emission tomography (PET), ultrasound, and magnetic resonance imaging (MRI). These techniques are often destructive, posing the risk of radiation ionization, and are often not compatible with patients with pre-existing conditions and medical devices such as pacemakers [78]. These are also expensive, involve sophisticated instruments, are time-consuming, and are often performed with multiple tests to avoid ambiguity [79]. Recently, there has been an increase in using plasmonic biosensing for cancer diagnosis and therapy, with review articles summarizing the progress in the same [80,81,82,83,84,85,86]. They are established to be minimally invasive, rapid, low cost, and offer point-of-care testing [87,88]. Of all plasmonic-based detection techniques, SERS is being extensively used for cancer identification, monitoring, and other theranostics, including imaging and chemo/photothermal therapy [89,90,91,92,93,94,95,96]. Figure 2 also indicates the growing interest in the last decade for the use of SERS-based plasmonic techniques for cancer diagnosis. SERS facilitates liquid biopsy [96] by using urine, saliva, and serum, thus making it low cost and enabling easier frequent sampling compared to the existing tissue-biopsy techniques, which are often destructive [97]. Different cancer biomarkers, such as miRNA [98,99], proteins, exosomes [100,101], circulating tumor DNA (ctDNA), genes [102], peptides [103], and blood plasma [104], are studied using SERS for disease identification. SERS tags that specifically bind to the targets under study are widely used for analyzing cancer samples [105,106,107,108,109]. Machine learning algorithms are used to analyze complex patterns and recognize buried signals overcoming noise from undesirable constituents of cells and other bio-fluids. Here, we focus only on SERS-based plasmonic biosensing for cancer-related applications in recent times, focusing on lung and breast cancers. 

#### 2.1.1. Lung Cancer

Lung cancer is known as the most fatal and frequently diagnosed cancer of all [110]. The cited report projected 2.89 million cases of lung cancer by 2030. Smoking, the presence of carcinogenic substances in the environment, and lifestyle are considered to be the main causes of lung cancer [88]. There are two kinds of lung cancers, non-small cell (NSCLC) and small cell lung cancer (SCLC). NSCLC is the most common kind of lung cancer, accounting for 80% of the cases. SCLC is the most fatal and fast-spreading cancer, and it is often diagnosed only at the later stages. SERS has been successfully used for the diagnosis of both kinds of lung cancers, with a prospect of developing point-of-care and rapid testing.

**Figure 3:**
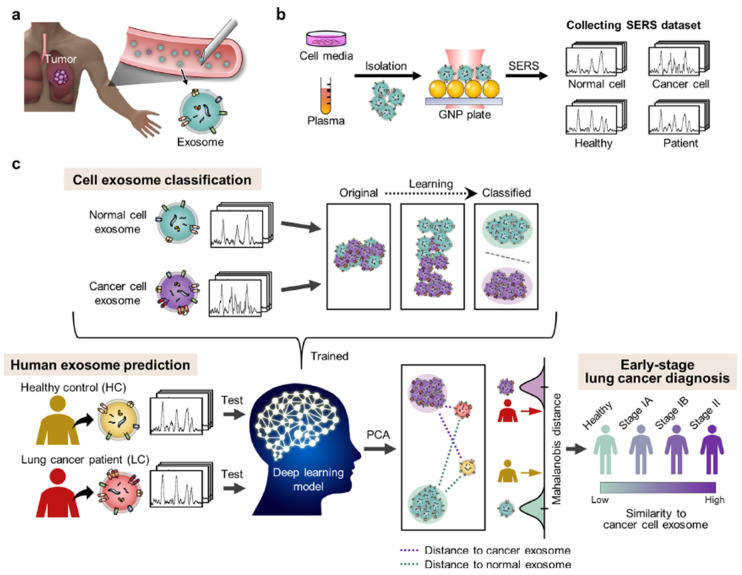
Schematic of work created by Hyunku et al. in lung cancer identification via a combination of SERS and deep learning. (**a**) Exosomes that were used as biomarkers for sensing. (**b**) Sample preparation and data collection. (**c**) Deep learning model used for the classification of normal and cancer cells exosomes using SERS spectra. Reproduced with permission from [118]. Copyright (2020), American Chemical Society.

Using DNA-based complexes as SERS tags and miRNA as a biomarker, Mao et al. have developed a lateral-flow-assay-based SERS substrate for the rapid detection and quantification of lung cancer biomarkers in less than 30 min [111]. Two types of biomarkers, miR-21 and miR-196a-5p, were detected simultaneously and with comparable accuracy with the existing qRT-PCR techniques. Similar studies were carried out with a flexible filter paper substrate [112] and with different biomarkers [113,114]. They extended their studies with ctDNA as the biomarker, thus implying the versatility of SERS [115]. Similarly, miRNA has been used for the detection of lung cancer by using circular exponential amplification reaction (EXPAR)-based SERS [116]. With a combination of asymmetric PCR and SERS, in regard to mutation genes in ctDNA, Guo et al. achieved a highly specific (100%) and sensitive (75%) lung-cancer-detection method in blood samples. Asymmetric PCA was performed to obtain single-stranded DNA, followed by the SERS-based detection using specifically labeled Au substrates [117]. With exosomes as biomarkers, lung cancers at different stages were identified accurately (~90%) by using SERS and a deep learning algorithm to classify healthy and malignant samples, exhibiting potential for early diagnosis, as shown in Figure 3 [118]. Serum samples of normal and lung-cancer patients were analyzed using PCA and PLS analysis to discriminate and identify the cancer samples with SERS spectra and achieved an accuracy of 92% [119]. Similarly, using a core-satellite type of plasmonic materials and SERS, serum samples of healthy, benign, and malignant cases of lung cancers were classified with a combination of principal component analysis (PCA) and support vector machines (SVMs) [120]. CtDNA-based identification of lung cancer using a DNA-rN1-DNA-mediated SERS frequency shift method was developed to achieve sub-femtomolar sensitivity [121]. Similarly, exosomes derived from bronchoalveolar fluid were used for the detection [122]. Whole-exosome SERS spectra have been analyzed using PCA to classify lung cancer and normal samples with 95.3% sensitivity and 97.3% specificity [123]. Lung-cancer biomarkers (aldehydes) and cells were identified rapidly using renewable porous CuFeSe_2_/Au nanostructures achieving an LOD of 1 ppb [124]. Challenging gaseous biomarkers called volatile organic compounds, which serve as indicators for lung cancer, were detected using ZIF-8-coated gold superparticles for the sensitive identification of lung cancer [125]. Pleural effusions of lung cancer and normal samples were studied using SERS and machine learning techniques to achieve a classification accuracy of 85% [126]. A combination of PCA and LDA has been used to classify lung cancer and normal samples with SERS analysis of serum samples, achieving sensitivity and specificity of 100% and 90%, respectively [127]. The same technique was used for SERS-based classification of lung-cancer-tissue slices [128]. Using common protein carcinoembryonic antigen (CEA) and a-fetoprotein (AFP) as biomarkers, SERS-based detection was performed for the diagnosis of lung cancer [129]. A non-destructive photothermal therapy targeting lung cancer cells (A549 cells) was developed using NIR radiation and Ag-Au shell–core structures were used for the SERS-based detection of the A549 cells. These nanostructures are highly specific and have different affinities for cancerous and non-cancerous cells, thus helping in tagging the cells. Based on the SERS activity of R6G molecules, the detection and phototherapy can be monitored [130]. Similar studies were carried out using reduced graphene oxide plasmonic substrates [131]. A multivariate analysis (SVM and PCA) of SERS data was used to identify and classify different types of lung cancers with an accuracy of 95% [132]. Choosing aldehydes in exhaled breath as biomarkers, highly sensitive, portable detection was performed, achieving LOD of 1.35 nM [133]. Chemometric techniques coupled with slippery liquid-infused porous surface-enhanced Raman spectroscopy were used for concentrating blood samples in a small area and thus enhancing the SERS signal for trace detection [134]. A gap mode plasmonic SERS substrate with a combination of Ag nanocubes and Au nanorods was used for the identification of lung-cancer-related exosomes [135]. A SERS analysis of saliva samples was performed to classify healthy and cancerous samples, using SVM and random forest, with a sensitivity of 95% and 97%, respectively [136]. With adenosine as a biomarker, urine samples were analyzed using SERS with Fe_3_O_4_/Au/Ag-based substrates, achieving good reproducibility, stability, and sensitivity of 10^−10^ M [137].

#### 2.1.2. Breast Cancer

**Figure 4:**
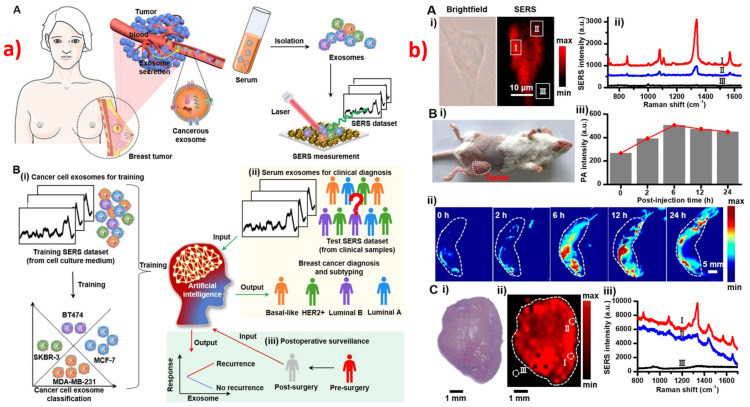
Breast cancer detection using SERS. (**a**) Schematic of workflow for (**A**) sample collection and (**B**) deep learning model-based breast cancer detection with exosomes-based SERS sensor. Reproduced with permission from [143]. Copyright (2022), American Chemical Society. (**b**) SERS and Photoacoustic (PA) imaging of breast cancer cells (**A**): (**i**) brightfield microscopic images and SERS mapping area, and (**ii**) corresponding SERS spectra in different regions labeled in (**i**). (**B**) (**i**) Photo of mouse with tumor, (**ii**) corresponding representative PA images for different post injection times, and (**iii**) PA intensity at 750 nm. (**C**) (**i**) Optical image of tumor, (**ii**) SERS image of tumor, and (**iii**) corresponding spectra for different regions in the image. Reproduced with permission from [144]. Copyright (2021), American Chemical Society.

Breast cancer is considered to be the second leading cause of death among women after lung cancer [138]. Breast cancer is often diagnosed by a mammogram, ultrasound, MRI, or biopsy. Furthermore, it is often concluded by a histopathological test, which is unfortunately time-consuming and is highly prone to human interpretation error. In addition to identification using urine, serum [139], and tear samples [140], SERS has also been used to understand the drug carrier mechanism [141] and classification of different stages of breast cancer [142], as shown in Figure 4.

Using the epidermal growth factor receptor as a biomarker, a gold-nanorods-based SERS tool that can identify and image the spatial and temporal distribution of breast cancer cells was developed by Xiao et al. [145]. Sialic acid, with its specificity towards a phenyboronic-acid-based nanoprobe, was used as biomarker for identification and imaging of breast cancer in human cells and saliva [146,147]. The miRNA of breast cancer was detected with a high sensitivity of 10^−10^ M, using a hybrid SERS substrate of GaN nanostructures with Au/Ag [148]. Functionalized SERS substrates with specific tags were used for the simultaneous isolation and detection of breast cancer cell lines [149]. Zheng et al. developed a SERS-based microfluidic channel for detection and quantification of prominent breast cancer biomarkers in real samples [150]. A combination of SERS and electrochemical biosensor was developed to monitor the drug response of DNA associated with breast cancer cells [52]. Hameed et al. have worked on fabricating anisotropic gold nano-stars that showed specific affinity to breast cancer cells compared to normal cells, aiding in the detection of the same [151]. Multiple SERS tags were used for understanding the drug-carrier mechanisms in breast cancer cells for the antineoplastic drug tamoxifen [141]. Similar studies were carried out with estrogen receptor alpha (ER-α) as the biomarker [152]. ER-α-based SERS has also been used for understanding cellular uptake mechanisms in breast cancer [153]. Labeled hollow silica-encapsulated gold nano-spheres were used for identifying and quantifying breast cancer biomarkers [154]. Choi et al. developed SERS nanotags by using Ag-Au hollow nanospheres that are durable, reproducible, and sensitive for the detection of various biomarkers for SERS [155]. A SERS-based 3D holograph was developed to detect and quantify nine miRNAs corresponding to breast cancer. Hairpin-like DNA was used as SERS tags along with Raman reporters for each miRNA that are spatially separated on the SERS substrate [156]. Similar studies were carried out by Weng et al. [157], Li et al. [158], and Lee et al. [159]. A ratio-type method was developed for the discrimination of breast cancer and non-cancer cells, using the SERS technique. A plasmonic material with Rh6G as a tag for breast cancer biomarker (MMP-2), along with a standard (2-NT), was used for analyzing the live cells based on the ratio of SERS signals in standard and R6G [160]. Li et al. also performed similar studies [161] and was also used for quantitative molecular phenotyping in a different study [162]. Recently, ratiometric SERS has been used for the identification of breast cancer using Au@Ag and GO nanostructures [163]. Cell suspensions of normal and breast cancer cells were analyzed using SERS coupled with Random Forest classifier to understand the differences. It was found that breast cancer cells have high cholesterol, lipids, proteins, and nucleic acids relative to the normal cells, and the classification accuracy was nearly 78% [164]. A comparison was made between SERS and Raman performance for the classification of different stages of breast cancers, using PCA and PLS-LDA, and found that SERS leads to better accuracy (94%) relative to the Raman (83%) method [142]. PLS-LDA was also used by Zheng et al. for the identification of breast-cancer biomarkers, using HAp [165]. PLS-SVM, PLS-LDA, and PCA-LDA were used for the classification of breast cancer and the normal group [166,167,168,169,170,171,172,173,174,175,176]. An exosome-based CNN model was developed for the classification of breast cancer and normal samples, with an accuracy of 95% [177]. A systematic analysis of SERS spectra obtained from urine and serum samples was performed, and it was found that the urine samples demonstrated better accuracy in the classification [170]. Biomarkers tracking the epithelial–mesenchymal transition in the plasma samples of breast cancer cells were identified using SERS immunoassay [178]. SERS-based cancer cell imaging was performed using gold nanoparticles based on the specific affinity of phenylalanine [179]. Different methods for the preparation of plasmonic Ag nanoparticles and their effects in SERS signal were discussed by Beata et al. [180]. Photothermal therapy and SERS-based identification of breast cancer were performed using gold nanorods [181] and gold nanobipyramids [182]. NIR and SERS-based phototherapy and detection were also performed [183]. A three-in-one tool consisting of photoacoustic imaging, thermosurgery, and SERS was developed to address the concern of residual microtumors in breast cancer [144]. By combining artificial intelligence and SERS, researchers developed a label-free detection method of breast cancer exosomes with 100% accuracy. This was also used to assess the outcomes of the surgeries [143]. Au/HCP-PS nanospheres were used for the SERS-based detection of breast cancer, using tears from asymptomatic patients, along with chemometric analysis [140]. A Pt-based SERS template was developed using cost-effective methods for the detection of breast cancer exosomes that achieved a sensitivity of 83.3% and a specificity of 95.8% [184]. A combination of 2D graphene and plasmonic gold nanostars was used for trace identification of exosomes [185]. There are many reports of researchers using exosomes as biomarkers for the identification of cancer, using SERS [186]. A highly sensitive (EF ~10^5^) and reproducible (2.7%) method was developed using Au@Ag nanospheres for the detection of breast-cancer-based extracellular metabolites [187]. Systematic experiments were performed to understand the effects of laser power and acquisition time on the reproducibility in immune-SERS microscopy and found that a longer acquisition time and higher laser power lead to poor reproducibility [188].

#### 2.1.3. Miscellaneous

With the mechanism for detection being the same, SERS has been extensively used for the detection of several other cancers, including gastric [189], oral, liver, ovarian [190,191], and prostate cancers [192,193,194,195,196,197]. Gastric cancer diagnoses have been performed using different plasmonic materials by analyzing SERS spectra of serum samples [198,199,200], blood plasma [201], exosomes [202], extracellular vesicles [203], telomerase [204], saliva [205], and ctDNA [206]. In a breakthrough study, a breath analysis based on SERS was performed to identify different stages of gastric cancers by analyzing the Raman bands [207,208]. Different chemometric techniques such as PCA [209,210], PCA-LDA [201,211,212,213], SVM [214], ANN [215], and PCA-QDA [216] were also used for classification and identification of gastric cancers. Li et al. used a combination of classification algorithms, such as PCA-LDA, PCA-SVM, and PCA-CART, for identifying gastric diseases in serum samples [217]. Blood samples from healthy and normal patients were analyzed for different cancers, such as liver cancer, colonic cancer, esophageal cancer, nasopharyngeal cancer, gastric cancer along with PCA-SVM and achieved an accuracy of 96% [218]. Based on the SERS profiling of urine samples, bladder cancer was studied using machine learning algorithms with miRNA as a biomarker [219]. PCA, random forest, KNN, and naive Bayes algorithms were used for the identification of renal cancer, with the SERS profiling of serum samples achieving accuracy greater than 75% [139]. Taking advantage of the coffee-ring effect, the serum samples of lung and prostate cancer patients were identified with 100% accuracy, using PLS-SVM algorithms on SERS data [220]. Gaussian-based CNNs were used for the same application elsewhere [221]. Recently, there was a review article specifically focusing on SERS-based biosensing for liver cancer detection applications [222]. Zhang et al. elaborated on the existing literature for oral cancer diagnosis and therapy with gold nanoparticles, highlighting the current progress and challenges [223]. A similar review article was also published for the case of ovarian cancer [224]. Oral cancer was studied using saliva samples and the miRNA of normal and cancer patients with the SERS technique [225,226]. SVM in combination with SERS has been used for the early detection of oral cancer among patients using serum and saliva samples and achieved an accuracy of 80% [227]. Prostate cancer has been extensively studied and successfully identified using different techniques, such as serum analysis combined with PCA-SVM [228]; detection of prostate specific antigens [229,230,231,232]; EVs combined with CNN [230]; miRNAs [233]; different multivariate techniques, e.g., PCA-LDA and PCA-SVM [234]; and urine profiling [235].

### 2.2. SARS-CoV-2 and Other Respiratory Diseases

With the onset of the pandemic and the fast-spreading variants, there was a need to rapidly identify, detect, and quarantine the infected population. Surveying the presence of antibodies in large populations, often called a serological survey, was important to access the percentage of population infected and to monitor community transmission [236]. The dominant existing technique for the identification of SARS-CoV was PCR, which relies on analyzing the genetic material of the virus [237]. However, the test is expensive, thus preventing wide usage and also is time consuming. The Raman spectrum of a whole organism, including viruses, is contributed to by the proteins, carbohydrates, and nucleic acids that make up the organism [238]. The expression of these building blocks is controlled by the genetic material of the organism, hence helping in the unique identification [239]. SERS has enabled trace, point-of-care (POC), sample-collection-friendly, rapid, flexible, and cost-effective covid detection alternatives with the use of diverse nanomaterials [10,240,241,242]. In addition, both portable and handheld systems have indeed enabled point-of-care testing based on Raman spectroscopy [56,243]. SERS has also been widely used for the detection of other respiratory zoonotic diseases, such as H1N1, H7N9, H3N2, and H5N1; and other coronaviruses, such as MERS-CoV [244,245]. Often, machine learning algorithms are used in combination to enable the identification of patterns that are not apparent to the human eye [246,247]. The availability of large data and the ease of collection have accelerated the potential of machine learning algorithms in identifying viruses and their variants with reliable accuracies for POC devices [248,249]. In addition to trace identification, SERS has also enabled quantification of viral load to access the severity of the infection [250,251].

**Figure 5:**
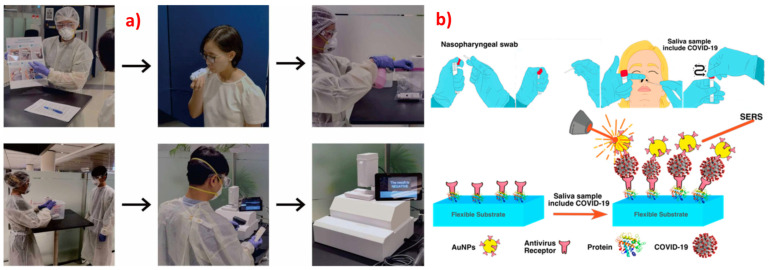
(**a**) Optical images of the workflow used for SERS-based detection of COVID-19 disease using breath analysis with a detection time of 5 min, achieving sensitivity >95% in nearly 500 participants, establishing the rapidness and specificity of SERS. Reproduced with permission from [53]. Copyright (2022), American Chemical Society. (**b**) Schematic of nasopharyngeal-swab-based Covid detection using SERS with flexible substrates enabling sensitive detection. Reproduced with permission from [238]. Copyright (2022), MDPI.

SERS in combination with LDA has been used for the rapid (2 min) identification of respiratory viruses, including SARS-CoV-2, human adenovirus type 7, and H1N1, using label-free silver nanoparticles [252]. Fe_3_O_4_@Ag nanoparticles tagged with specific antibodies were used for the detection of adenovirus and influenza virus [253]. Eleven different respiratory pathogens were identified using SERS, with nanoparticles tagged with nucleic acids achieving remarkable LODs in the sub-picomolar range [254]. Gold nanoparticles functionalized with a specific enzyme were used for the detection of S protein expressed by the COVID-19 viruses with SERS-based sensing in water [255]. Trace S protein detection has also been performed with SERS substrates enabling both chemical and electromagnetic enhancement [256] and using DNA-aptamer-based substrates, achieving a 0.7 fg mL^−1^ LOD [257]. Influenza-infected cells were identified based on proteins, using SERS and PCA [258]. Influenza and covid viruses were detected in human nasal fluid and saliva, using SERS [259], and also in untreated saliva [260]. A portable breath analyzer for covid detection based on the presence of organic volatile compounds was developed, achieving a sensitivity greater than 95% with less than 5 min of detection time [53]. A lateral-flow-immunoassay-based SERS was proposed for the quantitative detection of SARS-CoV-2 [261]. Similar work was performed for the trace detection of SARS-CoV-2 antibodies and spike proteins [262,263,264]. Li et al. optimized the silver nanostructures to increase the LOD for SARS-CoV-2 detection [265]. In a unique study, Kim et al. studied the efficacy of the Oxford–AstraZeneca vaccine by using SERS studies on tear samples and achieved excellent reproducibility and LOD in the femtomolar regime [266]. Machine learning algorithms such as PCA and SVM were used for the classification of normal and SARS-CoV-2 saliva samples with SERS data, with an accuracy of 95% [267]. Different respiratory viruses and their variants were identified using a silver-nanorods-based SERS sensor [268]. Different respiratory syncytial viruses have been identified and classified using SERS and classification algorithms such as PCA and HCA [269]. A deep-learning-based on-site SERS detection was developed to detect the SARS-CoV-2 virus based on the spike protein with 87% accuracy. This work also studied Raman modes of the spike protein theoretically and established a database [270]. Different variants of the SARS-CoV-2 virus, including wild-type, Alpha, Delta, and Omicron, were successfully identified using specific antibody-tagged 3D porous Ag-based SERS substrates [271]. SERS has also shown the potential of simultaneous detection of influenza virus (H1N1), SARS-CoV-2, and respiratory syncytial virus by using magnetic-tags-based SERS substrates with extended studies in throat swabs [272]. Label-free SERS was performed on serum samples of patients after 4 to 16 days of testing positive for COVID-19, and chemometric techniques were used to find significant difference in the SERS spectral features [273]. Figure 5 summarizes different techniques that are used for the SERS-based detection of SARS-CoV-2.

## 3. SERS-Based Detection of Microorganisms

### 3.1. Bacteria Sensing

A bacterium is a living cell and falls under the class of prokaryotic microorganisms. Bacteria come in different shapes, including spheres, rods, spiral, and comma, and have a typical size of few micrometers [274]. Bacterial cells are omnipresent, as they are found in water, food, soil, air, and the human body, and, interestingly, the human body contains 10 times more bacterial cells than human cells. However, only 3% of the bacteria are pathogenic, while the other 97% are essential for the survival of different life forms on the earth [275]. The identification of bacteria is important to assess the quality and contamination of food, soil, and water as a measure of public health. In some cases, the presence of bacteria is also desirable to ensure the decomposition of undesirable contaminants through a process called bioremediation [276,277,278]. Conventionally, PCR, plate culture, and flow cytometry are used for the detection of bacteria. However, all of them are time-consuming and need 2 to 3 days to arrive at conclusions [279]. SERS-based sensing for bacteria is extensively used for its proven advantages of being specific, sensitive [280,281,282], rapid [283], and water compatible to perform in situ measurements [284], as well as having the ability to quantify [285,286,287] and potential for trace detection [288,289,290,291,292,293]. Point-of-care devices for detection of bacteria can also be realized through SERS [294,295]. The sensitivity of SERS even enabled the detection of the single bacterium [295]. It is even possible to distinguish between live and dead bacteria cells by using SERS [296]. With the use of appropriate machine learning techniques, researchers achieved strain-level distinction using SERS spectra [297].

A SERS biosensor using aptamer (aptamer–Fe3O4@Au) and antibiotic (Vancomycin–Au@MBA) molecules has been used for the detection and quantification of pathogenic bacteria achieving a LOD of 3 cells/mL [298]. Vancomycin tagged NPs were also used in fabricating a sandwich such as SERS substrate for identification and photothermal elimination of bacteria in blood samples [299]. Different bacteria species such as *S. typhi*, *E. coli*, and *L. mono* were identified using SERS with Fe_3_O_4_@Au magnetic nanoparticles and demonstrated good accuracy in real world samples such as beef, saliva, and urine [300]. Wang et al. have also used magnetic nanoparticles for the detection of *S. aureus* [301,302]. Inspired by polyphenolic chemistry, SERS substrates with metal phenolic networks were designed for the detection of *E. coli* and *S. aureus* [303]. In addition to *E. coli* detection, antibiotic susceptibility was studied using core–shell Au@Ag nanorods. This study was also extended to mice blood, implying practical usage [304]. Bacteria present in serum and human blood sampl was identified using SERS based sensing [305,306]. Polymer mats prepared by force spinning were used for the detection of *S. aureus*, *P. aeruginosa*, and *S. Typhimurium* in blood plasma [307]. Using external magnetic field and plasmonic magnetic nanoparticles, the sensitive detection of Gram-negative bacteria was performed by concentrating the sample to a small area [308]. Similar work was accomplished using a microfluidic device to analyze drinking water for bacterial contamination [309]. The quantification of Salmonella typhimurium was performed using 3D DNA-based SERS substrates [310]. SERS-based immunoassay was used for the ultrasensitive and quantitative detection of different bacteria species simultaneously [311]. Multiplexing was also demonstrated by Hayleigh et al. [312] and Gracie et al., who then went on to conduct quantification in multiplexing [313]. A ceramic-filter-based SERS substrate, along with metal nanoparticles, was used for the detection of *E. coli* and Shewanella putrefaciens [314]. Nine different species of *E. coli* were studied using a SERS microfluidic device and discriminated with 92% accuracy, using support vector machine analysis [315]. The label-free and portable detection of various foodborne bacteria was studied using SERS and different chemometric techniques, e.g., PCA and PLS-DA [316]. Silver nanoparticles synthesized using leaf extract were used for the detection of two bacteria species [317]. SERS, in combination with deep learning techniques, was used for the accurate identification of Staphylococcus aureus to achieve an accuracy of ~98% [318].

### 3.2. Sensing of Biohazardous Molecules for Homeland Security

Bioterrorism is the new threat facing the world and is equally potent to cause large-scale destruction of civil, animal, and plant life. Often, biological agents are easy to prepare and scale up; can be contaminated in food, water, and soil; and are easy to carry, making them the future weapons. Many countries keep them in their military stockpiles despite the regulations [319]. According to the Centre for Disease Control and Prevention (CDC), a biohazardous material is defined as any infectious agent or biological material that poses a threat to human health, the environment, and animals. A review by Lister et al. summarized different biological agents that concern homeland security [320]. Different pathogens and biological agents, such as toxins, venom, and allergens, are some examples of biohazardous materials. Nerve agents are a big concern owing to their high solubility, high toxicity, and durability, with the Tokyo event in 1995 being an example [321,322]. Nerve agents can be classified into G-series, representing agents developed by Germans; V-series for venomous agents; GV series for the combination of G- and V-series; and Novichock series [323]. It is imperative to have a detection system that is sensitive, rapid, portable, and functional for different background media, such as liquids and gases, for the detection of these nerve agents. Plasmonic sensors are widely used for the detection of chemical and biological war threats [324,325]. Of all, SERS has its own advantages for the reasons discussed in the Introduction section and hence is widely used for the detection of biological threats, with a potential for field applications using portable devices [326] and chemometrics [327]. Here we focus on nerve agents, risk-grade-two and -three bacteria species, or their biomarkers’ sensing, using SERS, with an interest in homeland security.

A sensitive and selective identification of the nerve agents Tabun, Cyclosarin, and VX was performed using gold- and silver-coated Si nanostructures both without [328] and with a tag (antidote) [329] in two different studies. VX and its hydrolysis products were studied elsewhere, too [330,331]. Sarin, an organophosphorus nerve agent, was detected using plasmonic Si nanocone structures [332]. Three nerve agents, i.e., isopropyl methylphosphonofluoridate (GB), pinacolyl methylphosphonofluoridate (GD), and cyclohexyl methylphosphonofluoridate (GF), were identified, and their hydrolysis degradation was distinguished using SERS [322]. A mustard simulant, pathogenic bacteria, and cyanide were detected using SERS [333]. A reproducible (7%), rapid (30 s), and sensitive (1 ppb) was used for the detection of a nerve simulant, pinacolyl methyl phosphonic acid (PMPA) [334]. Gaseous warfare agents such as dimethyl methylphosphonate were identified using SERS on LiCl microlenses [335]. Various G-series and VX nerve agents were identified using novel pinhole shell-isolated Au nanoparticles substrates achieving sensitivity of 10 ng/L and 20 ng/L, respectively [336]. Using plasmonic 3D fractal structures, a G-series nerve agent called dimethyl methylphosphonate (DMMP) was detected in the gaseous state, with a sensitivity of 12 ppmV [337]. Bacillus anthracis is a highly infectious bacteria that causes the fatal disease anthrax in humans. It is a cause for concern because of its recent usage as a biowarfare agent by many countries [338]. Farrell et al. summarized different anthrax biomarkers and existing detection techniques [339]. Plasmonic metal decorated anisotropic Ni nanostructures were used for detection of dipicolinic acid (DPA), a biomarker for anthrax [340]. Specifically, tagged SERS substrates were used for the detection of anthrax protective antigens, achieving a remarkable LOD of 1 pg/mL [341]. A magnetic microfluidic SERS sensor using specifically tagged Au nanoparticles was used for the detection of the anthrax biomarker poly-γ-D-glutamic acid, with an LOD of 100 pg/mL [342]. Reusable and sensitive laser-ablated Au nanostructures were used for the detection of dipicolinic acid (DPA) with a LOD of 0.83 pg/L and signal enhancement of ~10^12^ [343]. A selective SERS substrate that can discriminate between different strains of bacteria by specifically binding to Bacillus anthracis was designed with DPA as a biomarker [344]. Gold nanorods were also employed for the sensitive detection of DPA and anthrax-protective antigen [345,346]. The trace detection of DPA, equivalent to nearly 18 spores, was achieved using super-hydrophobic SERS sensors [347]. The effects of aggregation of NPs and pH on the SERS performance for the detection of components of cell wall and endospores of Bacillus thuringiensis were studied extensively [348]. Different chemical and biological warfare agents were classified using techniques such as PCA, PLS-DA, as well as hierarchical classification techniques based on the SERS spectra [328,349].

## 4. Machine Learning in SERS-Based Biosensing

### 4.1. Introduction to Machine Learning

**Figure 6:**
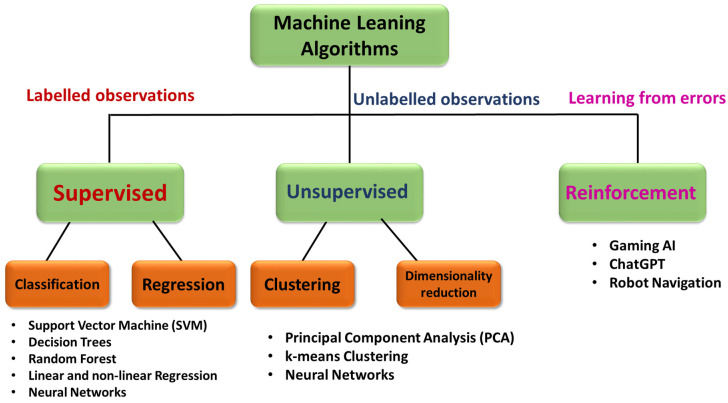
Flow chart illustrating the classification of different machine learning algorithms as supervised, unsupervised, and reinforcement models.

In recent times, machine learning is widely being used for many applications including spectroscopy for both data pre- and postprocessing. Machine learning (ML), as the name suggests, is a technique in which the algorithm learns patterns from the existing data and will attempt to make accurate predictions on the unknown based on the trained data. The potential for its ability to find complex patterns from big data sets has given an opportunity to extract and model data purposefully. There are different existing algorithms, both supervised and unsupervised, depending on the problem at hand. Deep learning is a subdomain of machine learning inspired by the human brain that uses multilayered neural networks for modeling data. Throughout this article, machine learning also implies deep learning techniques. Advances in computation facilities and with increasing availability and complexity of big data, deep learning, which is a kind of machine learning, has found its place. Some popular and relevant examples of ML being classification of emails as span and not span, identifying cancer in early stage using medical images, face recognition and weather prediction. ML algorithms can be broadly classified into three types, namely supervised for labeled observations, unsupervised for unlabeled observations, and reinforcement learning for models that learn from the errors to improve accuracy [350], as summarized in the Figure 6 below.

**Figure 7:**
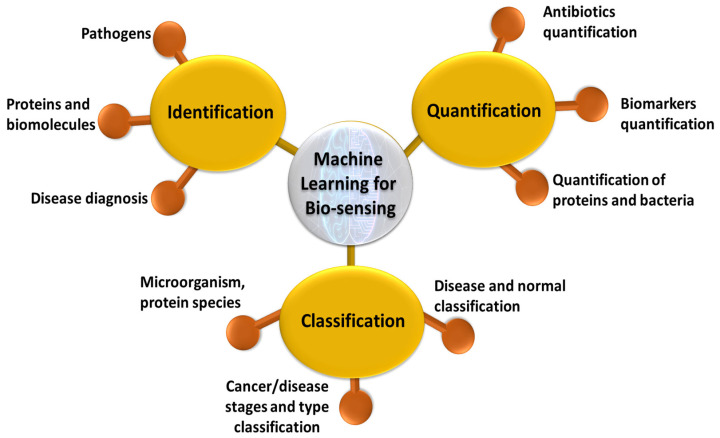
Schematic of applications of machine learning for biosensing using SERS based plasmonic sensors.

With the ease of data collection and availability of open source Raman spectroscopy data, SERS has also seen a surge in machine learning models [49,351,352]. The trend is welcoming and desirable as the nature of existing challenges in SERS involving trace detection, signal fluctuations, quantification and identification are complex with many variables calling for an analytical tool that has the ability to capture the patterns devoid of experts [353]. Trace detection implies identifying signal from a noisy background where ML could be aided. SERS is also known to have inherent signal fluctuations owing to localization of hotspots. Especially in the case of bio samples, they have background contribution from different undesirable components thus interfering with the signal and need ML algorithms to extract the useful information [2,354,355,356,357]. The process of data collection, identification of chemical composition and quantification is non-linear and is highly dependent on human intelligence making it a barrier to carry the benefits of SERS to onsite [358]. Some of the widely used techniques include Principal Component Analysis (PCA), Support Vector Machine (SVM), Partial Least Squares (PLS), Decision Trees (DTs) and Convolutional Neural Networks (CNNs). PCA is a dimensionality reduction technique where components representative of the data with large variance are preserved. This is extensively used a preprocessing step in order to reduce complexity of the models or also as a classification technique [359,360,361]. SVM is a nonlinear ML technique that can be used for both regression and classification [360]. It works by finding a hyperplane that distinguishes two or more classes using a kernel function [362]. If the data set is small and the number of variables is large, PLS is useful for its ability to still extract useful information and is often used for quantitative studies [363,364]. DTs are widely used for classification of the data using a method bootstrapping [365]. CNNs are a kind of neural networks which employ filters and pooled layers in the architecture and often used if the size of the data set is large enough and if images are involved in the modeling [366]. Specifically, in the field of biophotonics, machine learning models using SERS can be efficiently classified into three domains: identification, classification, and quantification, with interests such as disease and molecular diagnosis [367,368]; microorganism classification, identification, etc. [369,370,371,372]; and cancer diagnosis [373], as shown in Figure 7. In addition, machine learning was also used to improve data collection to overcome signal fluctuations and enhance the usability on site [374], to estimate the effect of scattering [375] and for the SERS signal enhancement itself [376]. In further sections, we discuss different ML techniques that were used in SERS for biology applications.

### 4.2. Identification

**Figure 8:**
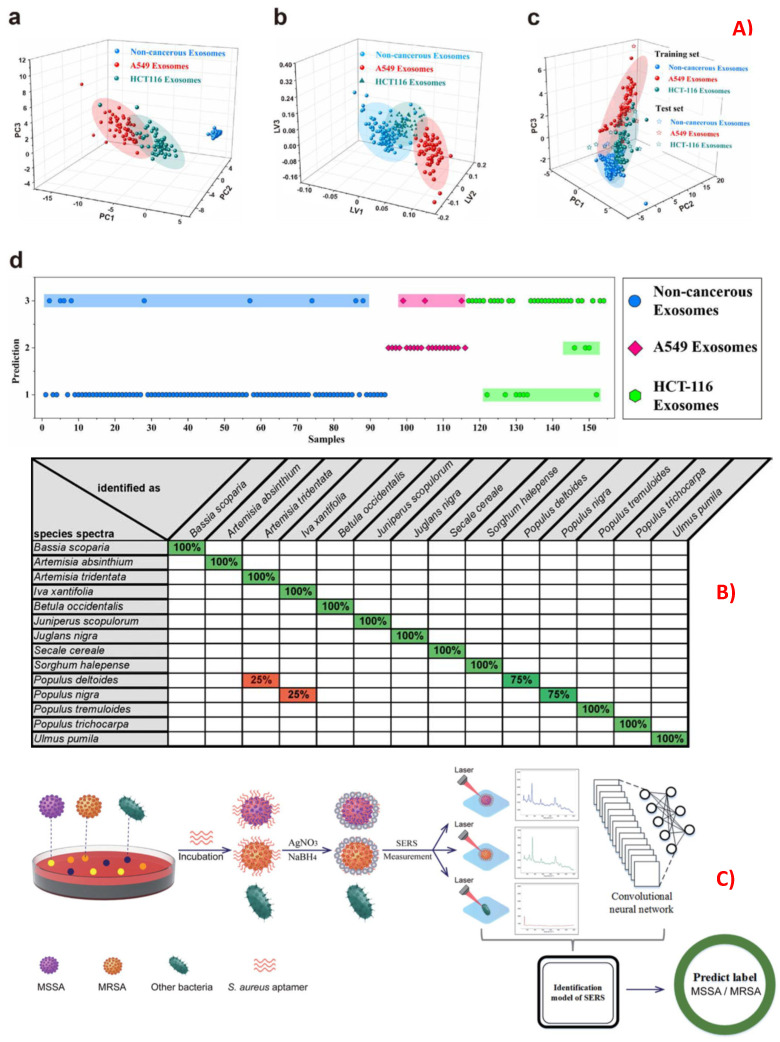
Applications of machine learning techniques used in the identification of biological samples, using the SERS technique for the (**A**) identification of lung and colon cancers from exosomes HCT-116 (colon cancer biomarker), A549 (lung cancer biomarker), and non-cancerous samples performed with (**a**) PCA, (**b**) PLSDA, and (**c**) SVM, with 60% of training set and 40% of test set. (**d**) Predicted labels for the test set using the SVM model. Value 1 is a prediction for normal plasma, Value 2 is a prediction for A549 (lung cancer) exosomes, and Value 3 is a prediction for HCT-116 (colon cancer) exosomes. The highlighted portion shows labels that are wrongly identified. Reproduced with permission from [382]. Copyright (2022), Elsevier. (**B**) Identification of 14 commercially available pollen species using SERS spectra combined with an artificial neural network, using a winner-takes-all (WTA) method. Reproduced with permission from [384]. Copyright (2015), Wiley. (**C**) Schematic for SERS-based ML model used in the identification of methicillin-susceptible Staphylococcus aureus (MSSA) and methicillin-resistant Staphylococcus aureus (MRSA) bacteria, using a CNN model. Reproduced with permission from [379]. Copyright (2021), RSC.

SERS provides the vibrational fingerprint of many biomolecules, including amino acids, peptides, carbohydrates, pathogens, and nuclei acids [377]. It is also label free and non-destructive, making it desirable for in situ and rapid identification. Often in real-world situations of biology sample analysis, there are undesirable effects from background cell signals or with the similarity of spectra from two subspecies. Machine learning models can be successfully trained to capture these complex differences and distinguish two similar spectra devoid of the background helping in identification of the sample. Figure 8 summarizes the work so far in using ML for identification applications in biosensing with SERS. CNNs were used for identification of cancer using SERS with gold multi-branched nanoparticles (AuMs), functionalized with different chemical groups, and achieved 100% accuracy in identifying the structural changes [378]. Drug-sensitive and drug-resistant bacterial strains were identified using SERS with a combination of CNNs and achieved 100% accuracy [379]. Different classification algorithms such as LDA, SVM, and KNN were used for the classification of bacterial extracellular vesicles for *E. coli* by strain and culture time using label-free approach of SERS [380]. SVM was successfully used for the identification of different drugs in human urine at trace levels with an accuracy greater than 92% [381]. A SERS chip was designed to identify a cancer marker, TIMP-1, and combined it with ML to identify lung and colon cancer in patients [382]. A label-free SERS, in combination with different machine learning algorithms, such as random forest, PCA-LDA, and decision trees, was used for the identification of colon cancer using serum samples. It was found that the random forest model outperformed the other two models in terms of accuracy and specificity [383]. SERS combined with ANN was used for the identification of different pollen samples despite many spectral contributions using Au NPs [384]. A microfluidic-chip-based SERS substrate with Au nanoparticles was used for the identification of l Jurkat, THP-1, and MONO-MAC-6 leukemia cell lysates, using SVM, and achieved 99% accuracy [385]. A lab-on-chip SERS device was fabricated and used for the successful identification of different species of mycobacteria [386]. The machine learning models PLS-DA and CNN were used to identify different stages of kidney malfunction in dialysis patients by using serum analysis by SERS. The CNN model achieved an accuracy of 96%, which is better than that of PLS-DA, with 84% [387]. The SVM outperformed other techniques in the identification of cyanobacteria, using SERS spectra of mutant and wild-type strains [388]. Using a dimensionality reduction technique, followed by a probabilistic ML model, SARS-CoV-2 identification was performed with an accuracy of ~85% [389]. SERS coupled with SVM was also used for the identification of lung cancers [96].

### 4.3. Quantification

One of the interests of using SERS for sensing also lies in its ability to detect trace and ultra-trace molecules. The intensity and concentration relation for a peak of choice in the SERS spectrum is often non-linear due to many factors, such as the inhomogeneous distribution of hotspots, non-uniform adsorption of molecules, and localization of the hotspots [361,390]. This calls for machine learning models that have the ability to capture non-linear patterns of intensity and concentration relation and further predict the unknown concentration. As the problem demands, regression ML models such as PCR, PLSR, SVR, and XGBR are used for the quantification of trace biomolecules.

**Figure 9:**
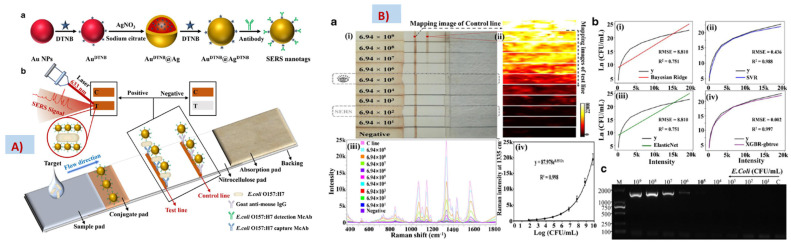
(**A**) Schematic of ML-based quantification of *E. coli* O157:H7, using (**a**) SERS nanotags and (**b**) lateral flow assay along with different regression models, including SVR, BNR, and XGBR. Reproduced with permission from [392]. Copyright (2020), Springer. (**B**) (**a**) (**i**) Optical images of the lateral flow strips and (**ii**) SERS mapping region of the prominent peak in the SERS intensity profile. (**iii**) Corresponding SERS spectra of the test lines. (**iv**) Intensity and concentration fit. (**b**) Machine-learning-based regression fits for (**i**) Bayesian ridge regression (BRR), (**ii**) support vector regression (SVR), and the (**iii**) elastic net regression (ENR) and (**iv**) extreme gradient boosting regression (XGBR). (**c**) PCR image for the *E. coli* detection. Reproduced with permission from [392]. Copyright (2020), Springer.

A quantitative analysis of antibiotics and a mixture of antibiotics was performed using PLSR with an accuracy of 96% [391]. An SERS-based lateral flow assay was used for the quantification of *E. coli* in milk and beef, using the Bayesian ridge regression (BRR), support vector regression (SVR), elastic net regression (ENR), and extreme gradient boosting regression (XGBR) algorithm, as shown in Figure 9 [392]. A SERS substrate with plasmonic nanogaps was fabricated and used for the trace sensing of pyocyanin, a secondary metabolite of Pseudomonas aeruginosa, from a complex background. Furthermore, using machine learning algorithms, the quantification of pyocyanin was performed with an accuracy until five significant digits, using PLS [393]. The quantification of very low concentrations of fumonicins in maize was performed using different chemometric techniques such as PCR and PLSR and achieved an accuracy above 90% [394]. Thiols found in the whole blood of umbilical cords were quantified using a PLSR model on SERS spectra collected using silver nanoparticles as plasmonic substrates [395]. PCA, followed by SVR, was used for the quantification of histamine, an allergen, in seafood, using spectral data from a combination of TLS and SERS [396].

### 4.4. Classification

The goal of the classification algorithms employed for data analysis in SERS for biosensing is often differentiating different classes, species, and spectra corresponding to different stages of the disease or different diseases themselves. So far, classification algorithms such as SVM, KNN, and PCA; and different neural networks, such as CNN, were used for the problems stated.

**Figure 10:**
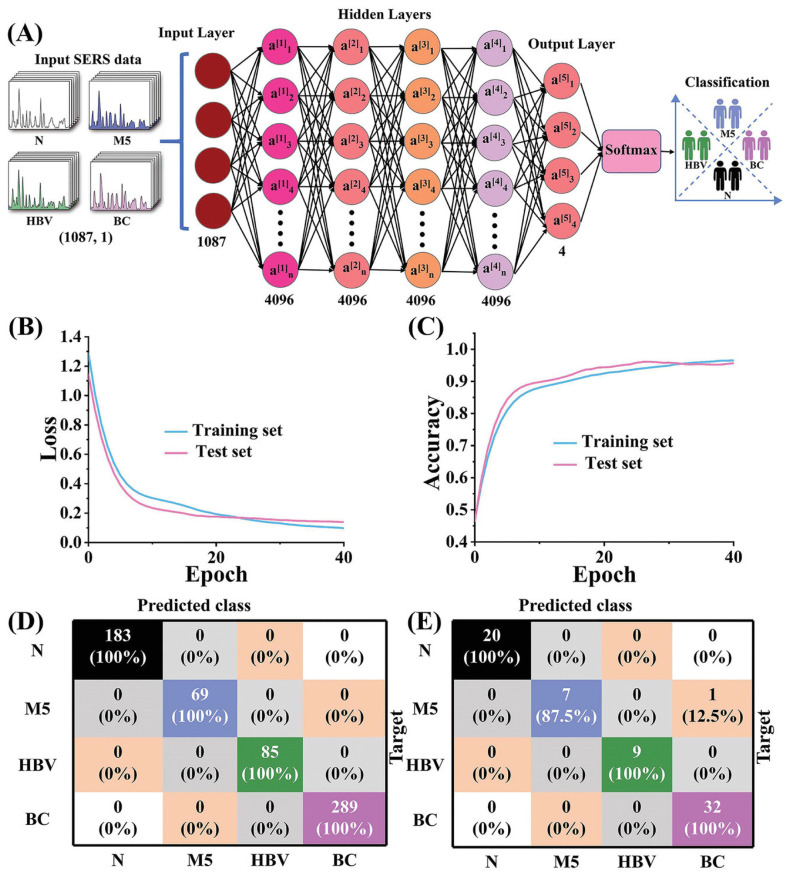
(**A**) Schematic of the architecture used for classification of different cancers and normal serum samples using SERS spectra collected from nearly 695 patients. (**B**) Learning curves for the model implemented with loss and (**C**) accuracy as metrics. (**D**) Confusion matrix for the training (**E**) test data sets communicating good accuracy. Reproduced with permission from [50]. Copyright (2021), Wiley.

Different bacteria species were classified and identified using SVM, with an accuracy of 87% by using SERS with bacterial cellulose nanocrystals (BCNCs) decorated with Au nanoparticles [397]. K-nearest neighbor and decision trees were used for the classification of SERS-based liquid biopsy assay to identify five protein biomarkers (CA19-9, HE4, MUC4, MMP7, and mesothelin) in pancreatic cancer patients, ovarian cancer patients, pancreatitis patients, and healthy individuals [398]. The direct serum analysis of liver cancer samples is performed using Au-Ag nano complex-decorated ZnO nanopillars on paper for the classification of different stages of cancer using CNNs. This method achieved an accuracy of 97.78% [399]. SERS combined with machine learning was also used for the screening of PCOS, using classification algorithms on SERS data. Samples of follicular fluids and plasma from healthy and PCOS patients were successfully classified, with an accuracy of 89%, using stacked models for both [400]. Protein species with similar spectral profiles were classified using principal component analysis (PCA) applied to SERS spectra [401]. CNNs without any preprocessing steps were used for the classification of different grades of bladder cancer tissue, using Raman spectra, and different species of *E. coli*, using SERS spectra. Different classification algorithms, such as KNN, PCA, SVM, and ANN, were used, but CNN was found to outperform the others in terms of accuracy [402]. Using Non-Structural Protein 1 (NS1) as a biomarker for dengue, extreme learning machine and PCA models were used for the classification of dengue patients with 100% accuracy towards a goal of early diagnosis [403]. Bacterial endotoxins of twelve different species were identified and classified using SERS spectra and machine learning algorithms such as KNN, RF, SVM, and RamanNet. While the other algorithms achieved accuracy greater than 90%, RamanNet outperformed them, with 100% accuracy [404]. With a goal to identify cancer at an early stage, a point-of-care diagnosis system using a novel hydrophobic SERS substrate combined with machine learning techniques was used, as shown in Figure 10 [50]. The SERS spectra of serum samples collected from nearly 690 patients, including normal and different cancers (breast cancer, leukemia, and hepatitis B virus), were collected and analyzed using deep learning techniques to achieve 100% accuracy in successfully classifying the data. They performed external testing with an accuracy of 98%, indicating potential usage in the real world.

## 5. Conclusions and Scope

The vast existing literature and continued interest in the SERS technique for biosensing is a promising sign to realize point-of-care devices based on SERS. This would revolutionize disease diagnosis due to its ability to identify traces, enabling early detection, cost effectiveness, and rapid diagnosis. Under optimized conditions, a single bacterial cell was also detected using SERS, thus demonstrating its sensitivity [295]. Using SERS, it is possible to identify disease biomarkers in a variety of bio-fluids, such as urine, saliva, plasma, and blood, as well as in volatile compounds and gases. Unlike many commercial techniques, SERS is reagent free and does not need sequential procedures for the identification of disease biomarkers. Machine learning techniques are extensively being used in SERS for their ability to recognize complex and intricate patterns devoid of background noise. Different models, such as PCA, SVM, ANN, CNN, KNN, and PLS, were used for identification, quantification, and classification of microorganisms and different diseases, including cancers. In regard to cancer diagnosis using SERS, the distinguishment between normal and cancerous samples, including cells and liquids, and the discrimination of different stages of cancer have also been performed. These methods are cost efficient, rapid, and sensitive, as opposed to the existing cancer-screening techniques. In response to the pandemic situation, SERS has been extensively used for the detection of novel COVID-19 virus and also for tracking the efficiency of the vaccines [266]. Furthermore, SERS has been widely used for the detection of various nerve agents and other bio-warfare agents, thus expanding its application in homeland security. Commercialization of SERS is already underway, with many lateral flow and point-of-care devices that have been developed in response to the pandemic [261] and diagnosis of other diseases with equal and par performance as existing commercial techniques [150]. For example, it is established that SERS performs better than the commercial enzyme-linked immunosorbent assay (ELISA) test kits in cancer detection, allowing multiplexing with very less sample volume [405]. It was also shown to achieve a lower LOD than radioimmunoassay (RIA) and ELISA in a different study [406]. In a recent study, SERS was compared with a clinically available method for quantification of glucose in blood sugar and shown to perform equally good [407]. It was found that SERS is 16-to-32-times more sensitive than the commercial lateral flow assay and >400-times more sensitive than the ELISA with the same reagents for the detection of covid [264]. The major components of a Raman system consist of a laser source, a probe for excitation and signal collection, and a detection system with a spectrometer [408]. Recent advances on all of these fronts for miniaturizing and reducing the cost are enabling the widespread usage of SERS-based detection with portable systems.

**Figure 11:**
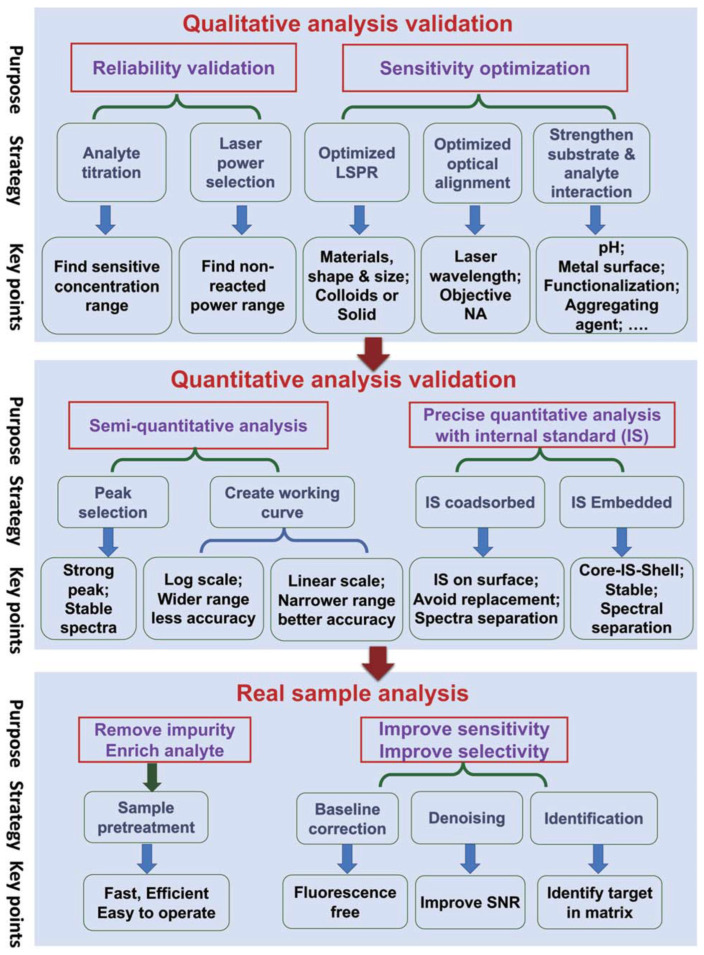
Steps for best practices for quantitative and qualitative detection using SERS to overcome the challenges at each stage with a goal of real-world applications. Reproduced with permission from [390]. Copyright (2020), RSC.

Despite its merits, there are few challenges that stand in the way of scaling up SERS for biosensing in the real world. Firstly, there are reasons inherent to the SERS enhancement mechanisms that turn out to be undesirable, often causing signal fluctuations and poor reproducibility. Due to the localization of dense field enhancement areas (“hotspots”) and metal-sample adsorption artefacts, SERS signals are known to fluctuate. Upon laser illumination, these hotspots are also known to diffuse or transform, thus adding further to the poor reproducibility. A substrate with homogeneous field enhancement promises good reproducibility but comes at the cost of enhancement and eventually limiting trace detection [374]. Secondly, SERS substrates majorly comprise Au or Ag nanoparticles/nanostructures. These nanostructures are generally not stable for long durations, with a risk of rapid oxidation upon exposure to ambient atmosphere. Owing to their large surface charge, they also tend to aggregate to form clusters. Often, aggregation and oxidation are prevented by the addition of capping agents or ligands which could affect the SERS signal and compatibility with the bio-samples. In view of commercialization, there is also a question of the reusability of the SERS substrates. Thirdly, SERS substrates that are used in the lab are optimized under specific instrument conditions, such as laser wavelength, acquisition time, power, and focusing conditions. In regard to point-of-care applications, it is a challenge to have the same experimental conditions, thus limiting the substrate efficiency. Field applications also call for cost-effective and miniature devices that are easy to operate by a non-expert [39]. Specifically in regard to biosensing, SERS-based detection in the field is a challenge because of the lack of disease specificity. In most of the scenarios, the biomarkers for disease detection, such as proteins and antigens, are not disease specific and need further evaluation in order for researchers to arrive at conclusions [409]. In the case of whole-organism or cell/tissue studies, it is difficult to ascertain the peaks because of contribution from various components, such as proteins, DNA bases, lipids, and other cell components. Figure 11 summarizes some best practices for quantification and qualitative analysis in SERS at various stages of experiments. The current research in SERS for biosensing is moving in the right direction to overcome these challenges with exploration in the direction of instrumentation, standoff detection, and the usage of ML techniques to improve data collection and identification without expertise. We believe that, in the coming years, these challenges will be successfully met and SERS will realize its full potential in real-world low-cost biosensing.
